# Tracheal adenoid cystic carcinoma mimicking a thyroid tumor: A case report

**DOI:** 10.3892/ol.2014.2282

**Published:** 2014-06-25

**Authors:** WOJCIECH KUKWA, PIOTR KORZEŃ, PIOTR WOJTOWICZ, GRZEGORZ SOBCZYK, DOROTA KIPRIAN, ANDRZEJ KAWECKI, ANDRZEJ KUKWA, ANTONI KRZESKI, CEZARY SZCZYLIK, ANNA M. CZARNECKA

**Affiliations:** 1 Department of Otolaryngology, Czerniakowski Hospital, Medical University of Warsaw, Warsaw 00-739, Poland; 2 Department of Oncology, Military Institute of Medicine, Warsaw 04-141, Poland; 3 The Maria Sklodowska Curie Memorial Cancer Centre and Institute of Oncology, Warsaw 02-781, Poland; 4 Department of Otolaryngology and Head and Neck Disease, University of Varmia and Masuria School of Medicine, Olsztyn 10-561, Poland

**Keywords:** adenoid cystic carcinoma, thyroid tumor, cylindroma, long-term remission

## Abstract

At present, only eight cases of tracheal adenoid cystic carcinomas (ACCs) mimicking thyroid tumors have been reported. Since there are no guidelines available regarding their diagnosis and treatment, they present a significant clinical challenge. In the present study, patient treatment was analyzed to deliver the first concise summary of treatment options in patients with ACC mimicking a thyroid tumor. In addition, all available data regarding molecular abnormalities of this disease have been discussed. The current study presents a case of a 17-year-old patient with a tracheal ACC mimicking a thyroid tumor. The patient was diagnosed in 2007 with a pathological mass between the left lobe of the thyroid and the trachea, and underwent surgery and radiotherapy. In 2010, multiple lesions in the lungs were diagnosed and pulmonary metastasectomy was performed. Following surgery, the patient has been disease-free for almost 30 months. Thyroid tumor biopsy may reveal ACCs. This pathological report requires further investigation of the head and neck in order to confirm if the disease is of tracheal origin. Patients may present with a neck swelling, hoarseness of voice or dysphagia. Surgery must be considered as first-line therapy for all patients with local disease as it may be curative. For palliative treatment chemoradiotherapy based on cisplatin may be effective. The identification of cytogenetics, tumor suppressor genes, oncogenes, epigenetic alterations and mitochondrial abnormalities specific for ACCs is critical to the development of targeted therapies. Thus far, large studies have only reported the transcriptional activator Myb and mammalian target of rapamycin signaling pathway to be disrupted in ACCs.

## Introduction

Tracheal neoplasms occur rarely, with an incidence rate of ~0.1 individuals per 100,000 population worldwide. Although they account for <1% of all reported malignancies, >80% are malignant. The majority of tracheal neoplasms are squamous cell carcinomas (SCCs). Only 10% are adenoid cystic carcinomas (ACCs; cylindromas). Furthermore, the majority of ACCs are diagnosed in middle-aged individuals, with no gender predilection, and not in children and juveniles (1). ACCs arise from the mixed seromucinous glands present in the tracheobronchial submucosa. ACC is a rare tumor which predominantly affects the major and accessory salivary glands (2). An ACC is a locally invasive tumor, which usually spreads via direct extension, submucosal or perineural invasion. It may also give rise to distant hematogenous metastases, with >50% of patients exhibiting metastases as the primary diagnosis. Among these, pulmonary metastases are the most common; however, metastases to the brain, bone, liver, kidneys, skin, abdomen and heart have also been reported (3–6). There have been few reports of direct extension of an ACC of the laryngotracheal complex to the thyroid, with clinical manifestation as a thyroid tumor; thus far, only eight cases have been reported (7). In those patients, the tumor most frequently involved the cricoid ring, the larynx and the subglottic area. In patients with tumors in the lower area of the trachea, the tumor was found to primarily invade the lungs, and laryngeal involvement was rare (8).

Previously, basaloid squamous cell carcinomas have frequently been confused with ACCs and mucoepidermoid carcinomas of the upper aerodigestive tract in pathological investigations; however, different genetic abnormalities are now known to be responsible for the former. The defining molecular feature of ACCs is the presence of a recurrent chromosomal translocation, [t(6;9);(q22–23; p23–24)], with the fusion transcript involving the MYB genes (transcriptional activator Myb) and nuclear factor 1 B-type (9). ACC is positive for cytokeratins (CKs), CK8, CK14 and CK17, and mucoepidermoid carcinoma is immunopositive for CK8, CK14, CK17 and CK19. Carcinoembryonic antigen immunoreactivity and carbohydrate antigen 19-9 has been detected in 100 and 50% of adenocarcinomas, respectively (10). The expression of c-kit may not serve as a useful marker for predicting outcomes in ACC patients (11). Furthermore, elevated carcinoembryonic antigen serum levels have been found to decline following surgical resection and correlate with disease recurrence (12,13). The biological behavior of the ACC of the tracheobronchial tree differs from that of other tracheal neoplasms; however, little is known with regards to the molecular biology of the disease. A series of case studies in Taiwan (14) revealed that the overexpression of human epidermal growth factor receptor 2, tumor suppressor protein p53 and prostaglandin-endoperoxide synthase 2 (cyclooxygenase-2, COX-2) affects the prognosis of ACC patients. The increased expression of p53 and B-cell lymphoma 2, which regulate cell death (apoptosis), was noted in 90% of ACCs, whereby the majority of tumor cells (66–99%) were found to be immunopositive. However, an association between the overexpression of these factors and the histological types, clinical staging and survival was not identified until recently (15). Metastatic ACC cells demonstrate epithelial-mesenchymal transition (EMT) and sphere-forming abilities. These cells exhibit a high expression of EMT-related genes, including *Snail, Twist1, Twist2, Slug*, zinc finger E-box binding homeobox 1 and 2 (*Zeb1* and *Zeb2*), glycogen synthase kinase 3β and transforming growth factor β2 and stem cell markers (*Nodal, Lefty, Oct-4, Pax6, Rex1,* and *Nanog*), as well as differentiation markers (sex-determining region Y), Brachyury and α-fetoprotein (16). The identification of genomic, proteomic and metabolomic abnormalities that promote the development and progression of ACC is critical to the development of specific targeted therapies. Recently, the role of molecular analysis-based treatment was analyzed in a single case (17). As a result of immunohistochemical analysis the mammalian target of rapamycin (mTOR) pathway was found to be significant in the pathobiology of ACCs. It has already been confirmed that mTOR is a central protein involved in carcinogenesis in other tumors and thus a reasonable drug target (18). Administration of its inhibitors, such as everolimus, may prolong survival in certain types of carcinoma and has already been approved by Food and Drug Administration. mTOR inhibitors, including temsirolimus and everolimus (19) may also present a potential drug to be tested for the treatment of ACC (17). At present, the design of robust clinical trials based on translational studies is critical for determining novel treatment moieties with optimal dosing schedules, and combinations of such therapies with classical treatment. Further study in this challenging field requires multicenter cooperation to compile molecular data and to initiate prospective trials to determine the roles of promising novel agents (9). Patient provided written informed consent.

## Case report

**Figure 1:**
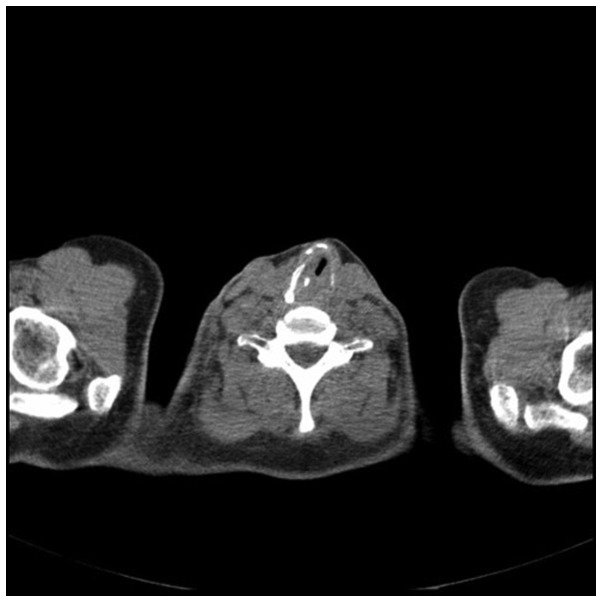
Postoperative computed tomography scan following resection of the tracheal tumor.

**Figure 2:**
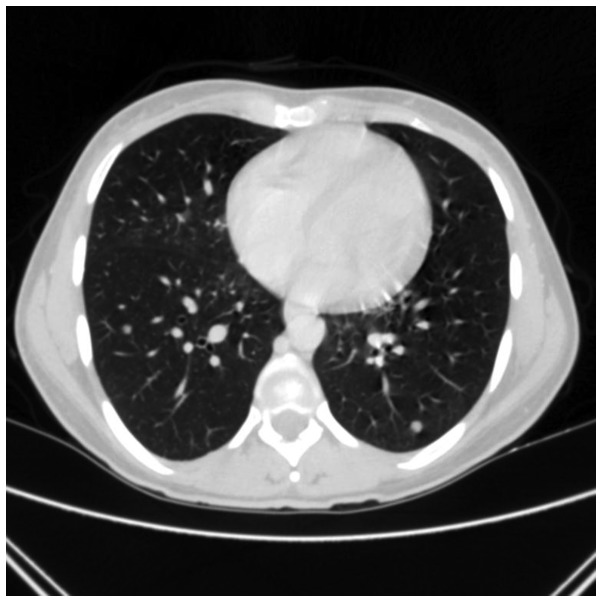
Computed tomography scan of the chest in axial section showing metastatic lesions.

**Figure 3:**
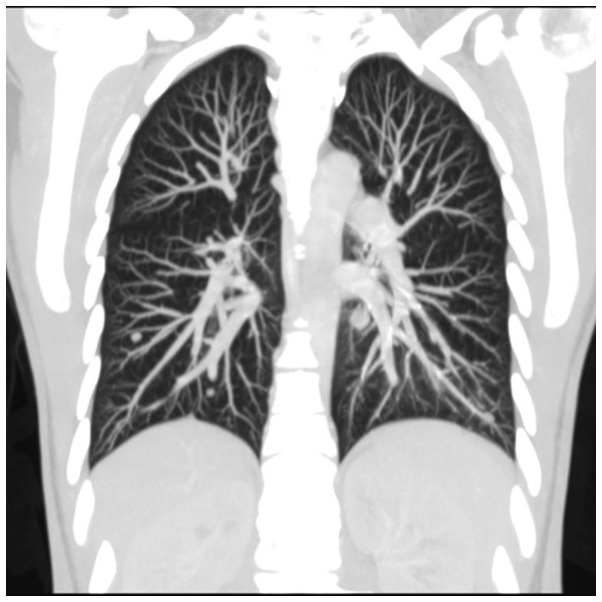
Computed tomography scan of the chest in coronal section showing metastatic lesions.

A 17-year-old female non-smoker with no significant past medical history presented with a non-productive cough, hemoptysis, dyspnea and breathlessness associated with wheezing for three months. The patient was diagnosed clinically with a pathological mass in the thyroid gland. In August 2007, ultrasonography was performed which revealed a 37×26 mm hypoechogenic lesion between the left lobe and the trachea, with enlarged hypoechogenic cervical nodes. Supra and infraclavicular lymph nodes were within normal size. Fine-needle aspiration biopsy was performed and indicated an ACC. Bronchoscopic examination revealed stenosis 1 cm in length under the patient’s vocal cords, with regular mucosa. A computed tomography (CT) scan confirmed a 34×37×50-mm tumor surrounding the trachea, infiltrating the two thyroid lobes and causing esophageal constriction. The cricoid and left arytenoid cartilage could not be assessed due to infiltration of the cancer. In November 2007, based on the results of imaging and pathological studies, the patient was referred to the Department of Otolaryngology at the Czerniakowski Hospital (Warsaw, Poland). The surgery plan included limited resection of the trachea, possibly extended with laryngectomy, and followed by thyroidectomy. In case of local invasion, segmental esophageal resection, with subsequent jejunal reconstruction was planned. The segmental tracheal resection was performed and completed with partial cricoid cartilage removal (Fig. 1). The procedure was completed with end-to-end anastomosis. In addition, thyroidectomy and tracheostomy finalized the surgical treatment. The postoperative histopathological examination confirmed the previous observations and revealed infiltration of the cricoid cartilage, muscles and fibrous sheath of the thyroid. No visible cancer cells were identified in the glandular tissue. Due to the microscopically positive surgical margin of the tumor, postoperative radiotherapy was performed with a fractional dose of 2 Gy and a total dose of 70 Gy. In August 2008, following postsurgical bilateral vocal fold paralysis, the patient underwent arytenoidectomy. In May 2010, a follow-up chest contrast-enhanced CT scan (Figs. 2 and 3) revealed multiple small metastatic-like lesions up to 13 mm in the lungs. The bronchofiberoscopy performed simultaneously ruled out neoplasm recurrence in the trachea. Therefore, in June and July 2010, the patient underwent bilateral thoracotomy, with marginal resection of the two lungs. Histopathological examination of the lung tissue confirmed metastatic disease. The patient was scheduled for routine follow-up. In April 2012, scheduled CT scans of the patient’s chest and neck revealed complete pathological remission. The patient has remained in a good condition since the removal of the tracheostomy tube in November 2012, and is able to eat without aspiration and does not require a gastric tube. Since the surgery, the patient has been treated with high-dose thyroid hormones and Ca^2+^ supplementation, and undergoes regular endocrinological evaluations. The patient has shown no signs of tetany. It is now more than five years since the diagnosis and more than a year since the pulmonary metastasectomy. The patient remains in a follow-up program and is in good health with a high quality of life. The patient undergoes scheduled oncological, laryngological and endocrinological consultations, and contrast-enhanced CT is used for routine imaging.

## Discussion

Rare types of cancer always pose a novel set of challenges. There are insufficient data to determine the best treatment approach for such cancers, including ACCs. Therapeutic decisions are based on small retrospective studies and case reports with limited periods of patient observation (20). In contrast to ACC of the salivary glands, the biological features of the tumors that develop from the tracheobronchial tree remain poorly understood. ACCs exhibit a predilection for perineural invasion and local and distant recurrences. The cancer cells of ACCs exhibit a high synthetic phase fraction, mitotic activity, vascular and lymphatic invasion, and often advanced tumor grading.

Generally, surgery must be considered as first-line therapy for patients with local disease, as it may be curative. Recently, it was suggested that surgical resection and primary reconstruction is the best curative treatment for primary tracheal cancer; however, in population-based studies this treatment is only applied in 10–25% of patients (21). Determination of the type of resection depends on the location of the tumor and its infiltration of other tissues. It may involve tracheal, laryngotracheal or carinal resection, with primary reconstruction or end-to-end anastomosis. The surgical approach includes transverse cervical incision for tumors restricted to the cervical trachea. Right posterolateral thorocotomy is reserved for lower parts of the airway tree; however, a combination of these methods may be used (22). For benign tumors, endoscopic resection and laser and electrocautery fulguration may be used as treatment options. The median survival time of patients with surgically resected tumors (68.8±1 months) is higher than that of patients with unresectable tumors (21.2±20.8 months). Furthermore, the five- and 10-year survival following resection varies from 59–79% and 29–51%, respectively (23). In addition, ≥27% of patients present with local recurrence 155±30 months following surgery. Distant metastases may occur in 55% of patients following a median time interval of 96 months (range, 24–180 months) from the initial surgery (24). The occurrence of adverse effects in the postoperative period is associated with the length of the resection, tension on the suture line, histological type of the tumor and the requirement for laryngeal release. The identification of surgery-associated risk factors is important due to a high mortality rate (≤10.8%) soon after surgery (22).

The function of radiotherapy in patients with ACC has been evaluated in several studies (25–27), with varying results, and its role remains uncertain. Neutron beam radiotherapy may be used for adjuvant therapy (28). Furthermore, ACC cells are less sensitive to ionizing radiation than SCC cells; however, this treatment must be recommended for all patients with unresectable disease or as adjuvant therapy. Patients with unresectable disease must be considered as candidates for definitive radiotherapy with a conventional photon dose of 80 Gy (26). In selected cases, the administered dose must be provided as five 2-Gy fractions per week over six weeks, with a total dose of 60–70 Gy. Intensity-modulated radiation therapy (IMRT) (29) or positron emission tomography CT-directed IMRT (27) may be employed rather than standard procedures. However, in contrast to SCC patients, studies have shown that ACC patients with node involvement who underwent complete resection may not receive any benefits from this treatment modality (23,30). Although radiotherapy alone is not recommended in the treatment of tracheal ACC, it may provide local disease control, and the majority of tumors respond to radiotherapy, which often results in long periods of remission (8).

If distant metastases are diagnosed, palliative treatment, including endobronchial treatment, low dose irradiation and best supportive care, are advisable (21). Surgery must be selected first whenever possible; however, for palliative treatment, radiotherapy, chemotherapy or chemoradiotherapy based on cisplatin may also be effective (23). Chemoradiation is a feasible treatment option and may lead to sustained locoregional tumor control in patients with non-resected ACCs. Surgical metastasectomy has also been reported as a useful treatment method (3–5). In a single case report, liver metastasectomy showed clinical efficacy (3). Pulmonary resections may result in locoregional control of primary disease and may extend disease-free survival (4,31). Endotracheal procedures, including forceps biopsy and suction, electrocoagulation, cryotherapy, laser, photodynamic therapy or argon-beam coagulation and stents, are used in palliative treatment to maintain upper airways patency (21).

Overall, ACC patients exhibit improved treatment outcomes when compared with SCC patients, with a five-year survival rate of 84 and 34%, respectively (31). However, it must be considered that although the five- and 10-year survival rates for ACC patients may be notable (79 and 57%, respectively), the long-term outcome is poor due to late local recurrences and late metastatic spread (24).

In conclusion, a tracheal ACC may present as a thyroid tumor. The present case report highlights the importance of detailed diagnosis of the tumors of the thyroid gland and detailed pathological evaluation of thyroid tumor specimens. In the current case, the patient’s disease course further clarifies the understanding of the metastatic course of ACC and confirms the importance of rapid metastasectomy. Finally, the analysis of this case contributes important information to the knowledge base regarding ACC treatment outcomes in young individuals. In general, it must be acknowledged that patients with tracheal ACCs may present with a midline swelling on their neck, without any respiratory complaints, hoarseness of voice or dysphagia, and a large, firm, non-tender, multilobular mass in the thyroid gland on physical examination. The differential diagnosis of primary tracheal ACCs includes basal cell adenomas, pleomorphic adenomas and basal cell adenocarcinomas. Complete surgical resection may lead to prolonged survival or complete remission. In young patients, anastomosis is feasible even when 6–7 cm of the trachea must be resected. Furthermore, subsequent oncological follow-up must be conducted for much longer than five years. No molecular markers have been found to be useful in predicting the progression of the disease or patient prognosis. Additional study of the molecular oncology of ACC is required in order to identify novel drug targets and develop effective treatment strategies.
